# Evaluation of Mutton Adulteration under the Effect of Mutton Flavour Essence Using Hyperspectral Imaging Combined with Machine Learning and Sparrow Search Algorithm

**DOI:** 10.3390/foods11152278

**Published:** 2022-07-30

**Authors:** Binbin Fan, Rongguang Zhu, Dongyu He, Shichang Wang, Xiaomin Cui, Xuedong Yao

**Affiliations:** 1 College of Mechanical and Electrical Engineering, Shihezi University, Shihezi 832003, China; binbfan@163.com (B.F.); hedy_1221@163.com (D.H.); scw_shzu@163.com (S.W.); xmincui@163.com (X.C.); yaoxuedong@126.com (X.Y.); 2 Key Laboratory of the Ministry of Agriculture and Rural Affairs, Shihezi University, Shihezi 832003, China

**Keywords:** food additive, mutton adulteration, near-infrared hyperspectral imaging, sparrow search algorithm, machine learning

## Abstract

The evaluation of mutton adulteration faces new challenges because of mutton flavour essence, which achieves a similar flavour between the adulterant and mutton. Hence, methods for classifying and quantifying the adulterated mutton under the effect of mutton flavour essence, based on near-infrared hyperspectral imaging (NIR-HSI, 1000–2500 nm) combined with machine learning (ML) and sparrow search algorithm (SSA), were proposed in this study. After spectral preprocessing via first derivative combined with multiple scattering correction (1D + MSC), classification and quantification models were established using back propagation neural network (BP), extreme learning machine (ELM) and support vector machine/regression (SVM/SVR). SSA was further used to explore the global optimal parameters of these models. Results showed that the performance of models improves after optimisation via the SSA. SSA-SVM achieved the optimal discrimination result, with an accuracy of 99.79% in the prediction set; SSA-SVR achieved the optimal prediction result, with an R_P_^2^ of 0.9304 and an RMSEP of 0.0458 g·g^−1^. Hence, NIR-HSI combined with ML and SSA is feasible for classification and quantification of mutton adulteration under the effect of mutton flavour essence. This study can provide a theoretical and practical reference for the evaluation and supervision of food quality under complex conditions.

## 1. Introduction

Mutton is a widely consumed meat around the world because of its high content of vitamins, proteins and trace elements, such as calcium, phosphorus and iron, which are needed by the human body [1,2,3]. Some unscrupulous merchants adulterate mutton with pork and additives to increase their profits because of the high price of mutton. Mutton flavour essence is a representative additive in the market that provides pork with a strong mutton flavour. This essence will release benzene, phenol and other toxic substances in the heating process that can cause serious harm to the health of consumers [4,5]. At the same time, the mutton flavour essence used for adulteration will seriously interfere with common discriminant results. At present, studies on the detection of mutton adulteration with pork under the effect of mutton flavour essence are lacking. This phenomenon harms both the interests and physical health of consumers [6]. Therefore, the evaluation of mutton adulteration with pork under the effect of mutton flavour essence is increasingly important.

Existing detection methods of meat adulteration mainly include sensory test, chromatographic analysis [7,8], immune analysis [9,10], DNA analysis [11,12] and electronic nose/taste [13,14]. Sensory tests have been unable to meet the need whilst other detection methods present high technical requirements and complex operation with the improvement in adulteration methods. In recent years, spectral technology has been widely used in adulteration detection of meat and meat products due to its simple and fast operation and non-destructive characteristics [15,16]. Near-infrared hyperspectral imaging (NIR-HSI) is a three-dimensional information acquisition technology combining spectral and image technology [17]. NIR-HSI has been applied to detect food adulteration, in particular, for meat products [18,19], such as pork lung in pork [20], jowl meat in pork [21] and ternary adulteration [22]. These reports mainly focus on pure meat adulteration. A variety of food additives are also mixed into meat to interfere with the detection because of the diversification of methods for meat adulteration. Note that meat detection under the effect of food additives has been reported. Nunes et al. [23] detected adulterated beef with multiple water-retaining agents based on mid-infrared spectroscopy and successfully distinguished adulterated beef. However, the detection of mutton adulteration under the effect of mutton flavour essence by hyperspectral means has not been reported. Hence, NIR-HSI was used to detect the adulteration of pork under the effect of mutton flavour essence in this paper.

The data analysis and modelling mainly depend on machine learning (ML) methods. Support vector machine (SVM), back propagation neural network (BP) and extreme learning machine (ELM) have been widely used in the detection of meat and adulteration [16,24,25,26,27]. The uses of new algorithms to optimise parameters and their combination with practical problems have become an important research direction for machine learning in recent years [28,29,30]. Quantum particle swarm optimisation (QPSO) was used to improve SVM in evaluating meat freshness [31]. An improved AdaBoost-BP model was applied to the rapid detection of mutton freshness [32]. Genetic algorithm (GA) was utilised to improve ELM for the identification of mutton parts [33]. Although these algorithms improve the performance of the model, they still present limitations, such as falling into the local optimal solution.

A swarm intelligence optimisation algorithm, named sparrow search algorithm (SSA), was proposed in 2020 to overcome the problem of traditional algorithms falling into the local optimum [34]. Compared with other common algorithms, SSA demonstrates the advantages of high precision, fast convergence, enhanced stability and robustness [34]. SSA was used for the optimisation of the model for detection [35,36,37]. SSA was used to optimise BP combined with hyperspectral means to detect protein content in milk rapidly; the results showed that its performance was superior to that of other methods [38]. However, the application of the SSA improved the model in meat adulteration, though detection remains unverified. Therefore, ML models are optimised by SSA for classification and quantification of adulterated mutton under the effect of mutton flavour essence.

In this study, the feasibility of near-infrared hyperspectral imaging combined with machine learning and sparrow search algorithm for evaluating adulterated mutton with pork under the effect of mutton flavour essence was explored. This study aims to: (1) acquire the spectral information of mutton, pork and adulterated mixture under the effect of mutton flavour essence using NIR-HSI; (2) establish classification models based on BP, ELM and SVM; (3) establish quantification models based on BP, ELM and SVR; (4) use SSA to optimise parameters in the classification and quantification models and select the optimum models by comparing the classification and quantification results.

## 2. Materials and Methods

### 2.1. Sample Preparation

**Figure 1:**
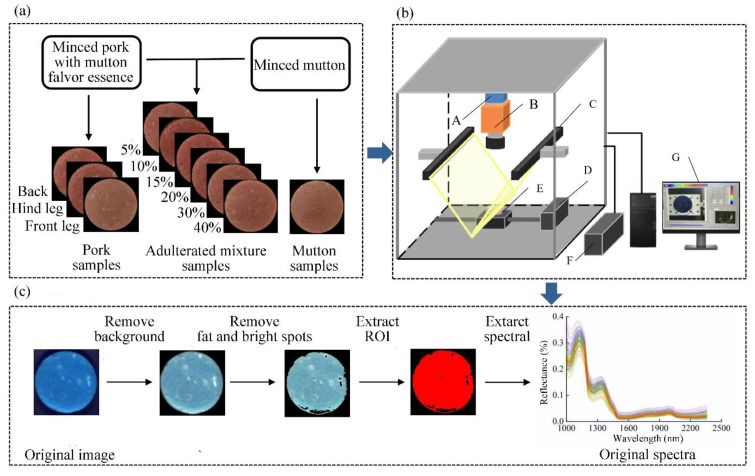
Schematic representation of sample preparation (**a**), NIR-HSI system (**b**) and spectral acquisition (**c**). (A) CCD camera; (B) imaging spectrograph; (C) light source; (D) electric moving stage; (E) sample; (F) light source controller; (G) computer and sample image. ROI: region of interest. NIR-HSI: near-infrared hyperspectral imaging.

Fresh mutton from the hind leg and fresh pork from three parts (front leg, hind leg and back) were used as meat sources in this study. The addition of different parts of pork can expand the scope of application and improve the generalization of the model. All meat was purchased from a local supermarket in Shihezi City, Xinjiang, and met national quarantine standards. The experimental meat was transported to the laboratory in an incubator at 4 °C. The processed meat was minced and mixed evenly with a meat-mincing machine (JYS-A900, Joyoung Co., Ltd., Zhejiang, China) for 30 s after the removal of evident fat and skin. The mutton flavour essence (Xianghaisheng Co., Ltd., Shandong, China) was weighed at a dosage of 3 g per kilogram of pork according to food safety regulations and then added into distilled water to prepare the solvent with a concentration of 0.05 g/mL. After that, the minced pork was added into the solvent and soaked for 20 min to mix fully. Then, the minced pork with mutton flavour essence was obtained after removing residue solvents on the surface. The minced pork from three parts with mutton flavour essence was mixed into the minced mutton in different proportions (5, 10, 15, 20, 30 and 40%) by weight to prepare the adulterated mutton samples. Fully mixed samples were placed in a Petri dish with a diameter of 6 cm for compaction and samples with smooth surface were obtained. A total of 144 adulterated samples were prepared for quantification, with 8 samples of each proportion in each part (8 × 6 × 3 = 144). Meanwhile, 24 pork samples from three parts (8 samples in each part) and 24 mutton samples were prepared using the same method. In total, 192 samples (24 + 24 + 144 = 192) in three categories were prepared for qualification. The prepared samples were stored in a refrigerator at −5 °C until detection. A diagram of sample preparation process is shown in Figure 1a.

### 2.2. Acquisition and Calibration of NIR-HSI Images

A liner scanning near-infrared hyperspectral imaging system was used in this study. It can produce 288 wavelengths in a spectral range of 1000–2500 nm. Figure 1b shows that the system consists of a spectrograph (ImSpector N25E 2/3, Spectral Imaging Ltd. Oulu, Finland), a CCD camera (Zephir-2.5-320, Photon Ltd., Montreal, Canada), a moving stage driven by a stepper motor, lighting sources (150 W), a computer and a monitor. Light intensity, exposure time, speed of the moving stage, illuminating angle of the light source and the height of lens were set to 100, 4 s, 62.77 mm/s, 60° and 170 mm, respectively, to obtain the real proportion of image size and high spatial resolution. Samples were placed on black cardboard as the background during the acquisition process. The experiment was carried out at 26 ± 1 °C and 30 ± 5% relative humidity.

The system was preheated for 30 min before image acquisition. Samples were then taken out of the refrigerator and left to warm to room temperature for 5 min. Sample images needed to be corrected with the standard black (with lens cover) and white reference (PTFE standard whiteboard) to reduce noise and external interference factors according to Equation (1):

(1) $$I=\frac{I_{R}-I_{B}}{I_{W}-I_{B}},$$

where *I* is the calibrated image data, *I_R_* is the original hyperspectral image data, *I_W_* is the full-white calibration image data and *I_B_* is the full-black calibration image data acquired after closing the camera with the lens cover.

### 2.3. Spectral Acquisition

The meat was separated from the background of the near-infrared hyperspectral image of the sample and the region of interest (ROI) was determined via image segmentation method [39]. ENVI 4.7 (Exelis Visual Information Solutions Ltd., Boulder, CO, USA) was used to process the original hyperspectral image and eliminate the interference. Firstly, backgrounds and shadows of samples were transformed to black using band subtraction and mask method. Fats and bright spots in samples were then highlighted through band addition and removed via mask method and binary processing. The pure muscle part of the samples was obtained after processing. Sample data were expanded to avoid under-fitting or over-fitting caused by insufficient data. Spectral data for each pixel were extracted according to the vertical direction of the ROI and randomly combined and divided into 10 blocks in equal proportion. The average spectrum of each block was extracted as the representative data of the sample. Lastly, 10 spectra were obtained for each sample and 1920 spectra were obtained for 192 samples. A wavelength of 1000–2445 nm was selected for analysis because the spectral noise after 2445 nm was high. The spectral acquisition is shown in Figure 1c.

### 2.4. Spectral Data Preprocessing

A certain amount of noise and interference information was generated due to external factors during the data acquisition [40]. Hence, preprocessing the original spectra is necessary. Spectral preprocessing can be divided into baseline correction, scattering correction, smoothing and scaling [41]. Baseline correction can eliminate the influence of instrument background or drift on signal. Scattering correction is used to remove the scattering effect caused by uneven distribution of particles. Smoothing can eliminate random noise and scaling can weaken data-scale difference. According to the characteristics of background interference and uneven distribution of sample particles in this study, the method of first derivative (1D) and multiple scattering correction (MSC) was selected as the spectral preprocessing method.

### 2.5. Model Establishment and Evaluation

#### 2.5.1. Modelling Methods

Back propagation neural network (BP) is a multi-layer feed-forward network algorithm based on error back propagation that presents high self-learning and adaptive ability [42]. It mainly realizes nonlinear mapping between input and output through learning. The number of hidden layers and the number of neurons in each layer can be set according to different situations and the performance varies with the structure.

Extreme learning machine (ELM) is a kind of machine learning method based on feed-forward neural network (FNN) and usually applied to supervised and unsupervised learning [43]. The weight of hidden layer nodes is selected randomly or artificially and does not need to be updated. The learning process presents advantages of strong generalization ability and fast calculation speed because it only calculates the output weight.

Support vector machine (SVM) is a supervised machine learning algorithm that determines the optimal separation hyperplane in the feature space to maximise the sample interval in the calibration set to avoid dimension disaster to some extent [44]. SVM can solve not only classification problems, but also regression problems through its important branch called support vector regression (SVR).

#### 2.5.2. Sparrow Search Algorithm Optimisation

Sparrow search algorithm (SSA) is a swarm intelligence optimisation algorithm for simulating the behaviour of sparrow foraging and evading predators [34]. SSA can effectively avoid falling into the local optimal solution because of its advantages of minimal iterations and high accuracy in the prediction model [45]. SSA was used in this study to optimise parameters of BP, ELM and SVM/SVR models. The process of constructing the optimisation model based on SSA is presented as follows.

Step 1: Initialise population, iterations, proportion of discoverer and participant, calculate and sort the fitness of all sparrows.

Step 2: Update the position of discoverers and participants. Discoverers with satisfactory fitness values can preferentially obtain food in the searching process and provide foraging direction for all participants. The participants will monitor the discoverers and immediately compete with discoverers when the discoverers search for better food in the foraging process. The position of the discoverers in each iteration is updated, as in Equations (2) and (3).

(2) $$X_{i,j}^{t+1}=\left\{ \begin{array}{l} X_{i,j}\cdot \exp(-\frac{i}{\alpha \cdot iter_{\max}}),if\;R_{2}<ST\\ X_{i,j}+Q\cdot L,if\;R_{2}\geq ST \end{array}\right.$$

(3) $$X_{i,j}^{t+1}=\left\{ \begin{array}{l} Q\cdot \exp(-\frac{X_{worst}-X_{i,j}^{t}}{i^{2}}),if\;i>n/2\\ X_{P}^{t+1}+\left|X_{i,j}-X_{P}^{t+1}\right|\cdot A^{+}\cdot L,otherwise \end{array}\right.$$

where *t* represents the current iteration number, *iter*_max_ is the maximum iteration number and *j* is the constant of [1, d]. *X_i,j_* is the position of the *i*th sparrow in the *j* dimension, *X_P_* is the best position for discoverers and *X_worst_* is the worst position. The discoverers can search in a safe foraging environment when *R*_2_ *< ST*. The predators appear and all sparrows move to forage rapidly when *R*_2_ *≥* *ST*. The *i*th participant with low-fitness value travels to other places for food when *i* > *n*/2.

Step 3: Update the position of watchmen. Watchmen are randomly generated in the population and generally account for 10% to 20% of the total. The position of the watchmen in each iteration is updated as in Equation (4).

(4) $$X_{i,j}^{t+1}=\left\{ \begin{array}{l} X_{best}^{t}+\beta \cdot \left|X_{i,j}^{t}-X_{best}^{t}\right|,if\;f_{i}>f_{g}^{}\\ X_{i,j}^{t}+K\cdot \left(\frac{\left|X_{i,j}^{t}-X_{worst}^{t}\right|}{\left(f_{i}-f_{w}\right)+\varepsilon }\right),if\;f_{i}=f_{g}^{} \end{array}\right.$$

where *X_best_* is the global optimal position; *β* is the step-size control parameter; *f_i_* is the current individual fitness value; and *f_g_* and *f_w_* are the global best and global worst fitness values, respectively.

In this study, the initial population size was 20; the maximum number of iterations was 100; the proportion of discoverers and participants was 7:3; the proportion of watchmen was set to be 20%. These parameters were determined according to the commonly used parameters and the actual situation in this study [46]. SSA was applied to optimise the model parameters and obtain the optimal solution from the global optimal position. The optimal solution for BP and ELM was obtained through iteration and assigned to weights and thresholds to complete the parameter optimisation of the SSA-BP and SSA-ELM models. The range of weights and thresholds is (−5, 5). The optimal solution for SVM and SVR was obtained through iteration and assigned to the penalty coefficient *c* and RBF kernel function radius *g* to complete the parameter optimisation of SSA-SVM and SSA-SVR models, respectively. The range of *c* and *g* is (1 × 10^−^^5^, 100).

#### 2.5.3. Establishment and Evaluation of Classification Models

In the establishment of classification models, 24 mutton, 24 pork and 48 adulterated mixture samples in each part (24 + 24 + 48 × 3 = 192 samples) were randomly divided into calibration set (144 samples) and prediction set (48 samples) at a ratio of 3:1. As such, 10 average spectra were extracted from each sample region. Hence, calibration and prediction set included 1440 and 450 spectra, respectively. Three classes were set: mutton (CLASS M), pork (CLASS P) and adulterated mixture (CLASS MP).

Firstly, ELM, BP and SVM classification models were established for different classes of samples. Secondly, SSA was used to find the global optimal position as the potential optimal solution of the models. Lastly, the SSA-ELM, SSA-BP and SSA-SVM models were constructed using optimal parameters. The 10-fold cross-validation divided the data set into 10 parts, of which 9 parts were used as training data and 1 part as test data in turn. It is a commonly used method to improve the generalization ability of the model. The 10-fold cross-validation result is taken as the calibration set result.

The performance of the models was evaluated using the discriminant results of each set after establishing the classification models. The discriminant property in the classification model was evaluated according to accuracy and accuracy confusion matrix. Accuracy is the ratio of the correct classification samples to the total samples. High model accuracy and few error classes in the confusion matrix correspond to enhanced model performance.

#### 2.5.4. Establishment and Evaluation of Quantification Models

In the establishment of quantification models, 8 adulterated mixture samples in each proportion of each part (8 × 6 × 3, a total of 144 samples) were randomly divided into calibration set (108 samples) and prediction set (36 samples) at a ratio of 3:1. As such, 10 average spectra were extracted from each sample region. Hence, calibration and prediction set included 1050 and 360 spectra, respectively.

Firstly, ELM, BP and SVM classification models were established for different proportions of samples. Secondly, SSA was used to find the global optimal position as the potential optimal solution of the models. Lastly, the SSA-ELM, SSA-BP and SSA-SVR models were constructed using optimal parameters. The 10-fold cross-validation result is the calibration set result.

The performance of models was evaluated by the regression results of each set. The regression property of the quantification models was evaluated with coefficient of determination (R^2^) and root mean square error (RMSE). High-R^2^ and low-RMSE values represent enhanced performance. The software used in this study was MATLAB 2019b (Math Works, Natick, MA, USA).

## 3. Results and Discussion

### 3.1. Analysis of Spectral Characteristics

**Figure 2:**
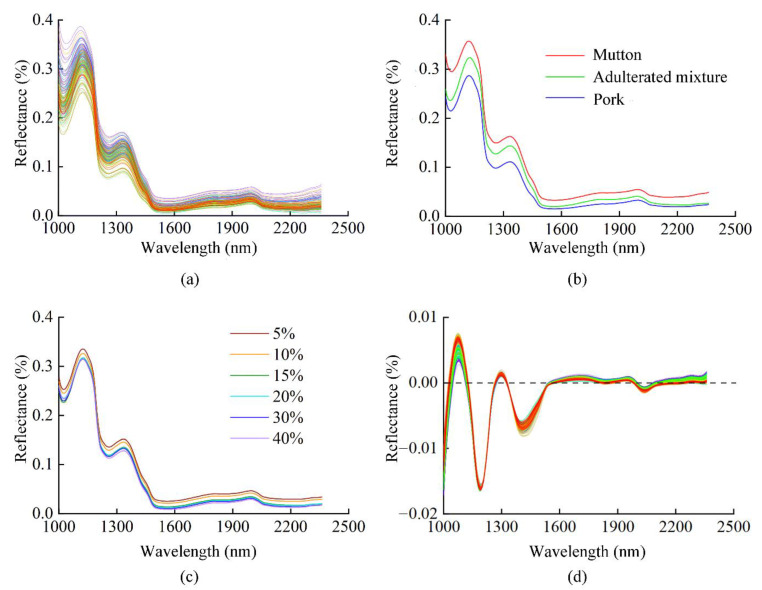
Spectra of samples. (**a**) Original spectra of all samples; (**b**) average spectra of different classes; (**c**) average spectra of different proportions; (**d**) spectra preprocessed with 1D + MSC.

The reflectance spectral curves of all samples are shown in Figure 2a. The spectral trends were similar and spectral absorption peaks mainly appeared at 1020, 1250, 1520 and 2150 nm. Amongst them, the spectral absorption bands at 1020 and 1520 nm were closely related to the stretching vibration of the first-order and second-order frequency doubling characteristics of the N-H bond, located at the functional groups of the protein molecular structure, respectively. The absorption peak at 1250 nm was related to the second-order frequency doubling characteristic in the C-H bond in the molecular structure of meat organic components. The absorption band at 2150 nm was related to the combination frequency characteristic in the O-H bond of molecular structure functional groups in water [47]. The region between 2200 and 2445 nm was mainly caused by the combined stretching vibrations of O-H, N-H and C-H functional groups, which showed low spectral reflectance [48].

The average spectral curves for mutton, pork and adulterated mixture are shown in Figure 2b. The average spectral curves for different classes of samples demonstrated the same trend but significantly different reflectance. This indicated that differences exist in the chemical composition and structural information between pork and mutton. The average spectral curves for adulterated mixtures of different proportions are presented in Figure 2c. Spectral tendencies of all adulterated samples are basically the same, with some differences in the reflectance. It revealed that different proportions of pork and mutton changed the composition content and material structure of the sample. The results show that the sample spectral information obtained in this study can distinguish amongst pork, mutton and adulterated mixtures, as well as different proportions of pork in mutton. This regularity was obtained under the effect of mutton flavour essence, thereby indicating that the spectral information in this study can avoid the interference of mutton flavour essence.

The original spectra were preprocessed using first derivative combined with multiple scattering correction (1D + MSC). The processed spectra are shown in Figure 2d. The processed average spectral curves of different classes and different proportions are shown in Figure S1 and S2 (Supplementary Materials). Compared with the original spectral curves, preprocessed spectra are more convergent, the influence of random noise is weaker and the reflection and absorption bands are more evident. The subsequent spectral data in this study were all preprocessed with 1D + MSC method.

### 3.2. Establishment and Evaluation of Classification Model

#### 3.2.1. Model Establishment

BP with three layers of network structure, including input layer, hidden layer and output layer, was used to establish the classification model. Traingdx training function and logsig and purelin transfer functions were selected. The number of hidden layer nodes was traversed in 5 steps from 5 to 100. The traversal results showed that the fitting effect was optimal when the number of hidden layer nodes was 20. ELM was also used to establish the classification model. Sigmoid function was utilised and the hidden layer was traversed in 10 steps from 10 to 200. The fitting was optimal when the number of neurons in the hidden layer was 60. At the same time, support vector machine (SVM) was applied to establish the classification model and grid optimisation was performed to determine *c* and *g*. The accuracy of the 10-fold cross-validation was used as the optimisation objective. The accuracy of the classification models in each data set is presented in Table 1. A comparison of the accuracy in the classification models established by BP, ELM and SVM demonstrated that SVM achieves the best modelling results. The accuracy of the calibration and prediction sets was 99.65 and 99.37%, respectively. The performance of SVM was clearly better than that of the BP and ELM models.

#### 3.2.2. Model Optimisation and Comparison

The SSA was used to optimise parameters *c* and *g* in the SVM model and was used to optimise the weights and thresholds of the BP and ELM models. The optimisation objective was the maximum accuracy of the 10-fold cross-validation. Optimal parameters were obtained and the models were established when the accuracy, as the fitness values, were the maximum. The results of SSA-BP, SSA-ELM and SSA-SVM are listed in Table 1. The BP, ELM and SVM models optimised by SSA exhibited satisfactory performance, especially the SSA-ELM and SSA-SVM models.

**Figure 3:**
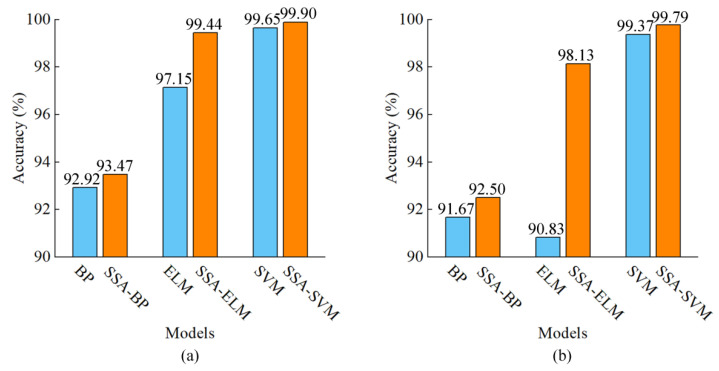
Comparison for accuracy of models in each data set. (**a**) Calibration set (10-fold cross-validation); (**b**) prediction set. BP: back propagation neural network; ELM: extreme learning machine; SVM: support vector machine; SSA: sparrow search algorithm.

The SSA-BP, SSA-ELM and SSA-SVM models were further compared with the BP, ELM and SVM models. The comparison of the accuracy of each data set before and after the optimisation is presented in Figure 3. The accuracy of each data set of the BP, ELM and SVM models optimised by the SSA was higher than that before optimisation. Amongst them, SSA-ELM showed the highest improvement compared with that before optimisation, followed by SSA-BP and SSA-SVM. The SSA-BP classification model was 0.55% and 0.83% higher than the BP model in the calibration and prediction set, respectively. The performance of the BP model slightly improved after SSA parameter optimisation. The accuracy of SSA-ELM increased by 2.29% and 7.30% in the calibration and prediction set compared with that of the ELM model, respectively. The SSA-ELM model evidently improved. The SSA-SVM model was improved with an accuracy of 99.90% and 99.79% in two data sets. Compared with the SVM model, the SSA-SVM model was 0.25% and 0.42% higher in the calibration set and prediction set. These results showed that the comprehensive classification performance of each model can improve when SSA optimisation is applied and the SSA-SVM model achieves an optimal result.

**Figure 4:**
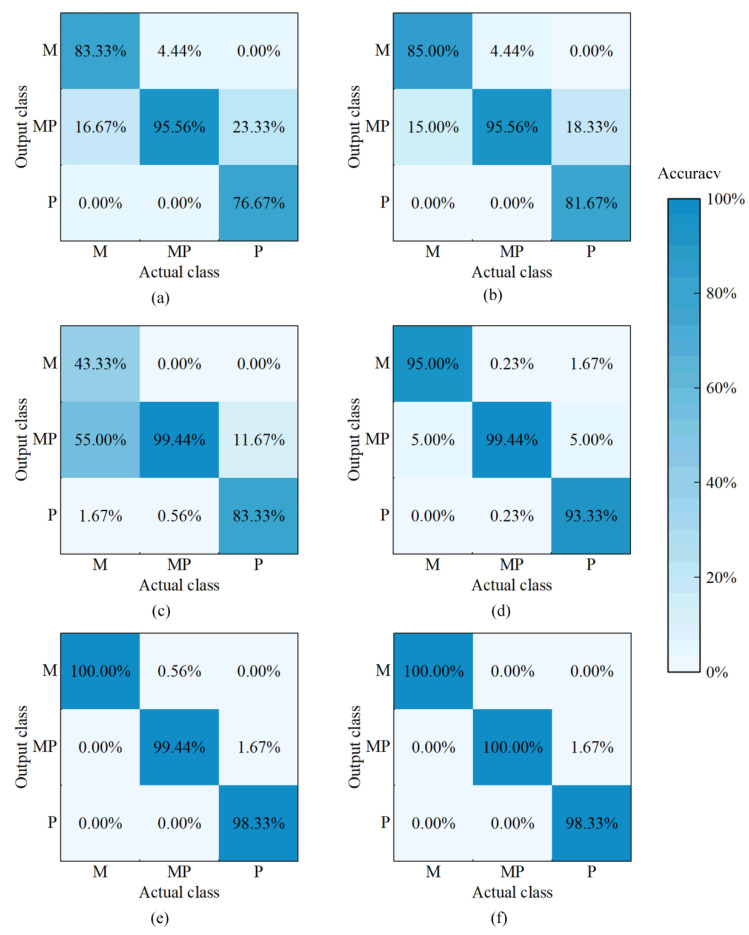
Confusion matrix of accuracy for different models in prediction set. (**a**) BP; (**b**) SSA-BP; (**c**) ELM; (**d**) SSA-ELM; (**e**) SVM; (**f**) SSA-SVM. M, P and MP represent mutton, pork, adulterated mixture, respectively.

In order to explore the classification performance of different models for each class of samples, the accuracy of the prediction set is illustrated as a confusion matrix in Figure 4. The single-class accuracy of different models improved after SSA optimisation. Amongst them, the accuracy of the BP model in discriminating mutton (CLASS M) and pork (CLASS P) was low, only 83.33 and 76.67%, respectively. The accuracy of SSA-BP increased to 85 and 81.67% when detecting CLASS M and CLASS P, respectively. Notably, the classification performance still needs to improve, despite showing a slight enhancement after optimisation. The ELM model was insufficient in discriminating CLASS M and CLASS P, with an accuracy of only 43.33 and 88.33%, respectively. The accuracy of SSA-ELM in detecting CLASS M and CLASS P increased to 95 and 93.33%, respectively. Although the improvement was evident, each class contained certain errors. The SVM and SSA-SVM can achieve a detection accuracy of 100% for CLASS M, which was significantly higher than that of other models. The detection accuracy of the SSA-SVM on adulterated mixture (CLASS MP) was the maximum at 100%. The detection accuracy of CLASS P was the highest at 98.33%. These results showed that SSA-SVM achieves the optimal classification performance for each class.

In summary, the performance of the SSA-BP, SSA-ELM and SSA-SVM models improved compared with that of the BP, ELM and SVM models, respectively. Amongst them, the SSA-SVM model obtained the best classification result. Although the mutton flavour essence changed odour information and effected sensory testing, NIR-HSI combined with the SSA-SVM model can effectively identify mutton, pork and adulterated mixtures with pork.

### 3.3. Establishment and Evaluation of Quantification Model

#### 3.3.1. Model Establishment

The quantification model for the adulterated mixture was established using BP, ELM and SVR. The optimisation objective was the error value of 10-fold cross-validation. The results of the models for predicting pork proportion in an adulterated mixture are listed in Table 2. The SVR model presented the best performance compared with the ELM and BP model. SVR demonstrated an R^2^ of 0.9290 and 0.8613 and RMSE of 0.0358 and 0.0699 in the calibration set and prediction set, respectively. The RMSE in the prediction set was much higher than that of the calibration set, which was close to twice. Therefore, the models need further optimisation.

#### 3.3.2. Model Optimisation and Comparison

SSA was used to optimise parameters *c* and *g* in the SVR model. Meanwhile, SSA was used to optimise the weights and thresholds in the BP and ELM regression models. The optimisation objective was to minimize the error values in 10-fold cross-validation. Optimal parameters were obtained and the SSA-BP, SSA-ELM and SSA-SVR models were established when error values were the minimum. The results of the three models are listed in Table 2. Amongst the models with SSA optimisation, the SSA-SVR model demonstrated the best result with an R_C_^2^ and R_P_^2^ of 0.9491 and 0.9304, respectively, and an RMSEC and RMSEP of 0.0335 g·g^−1^ and 0.0458 g·g^−1^, respectively.

**Figure 5:**
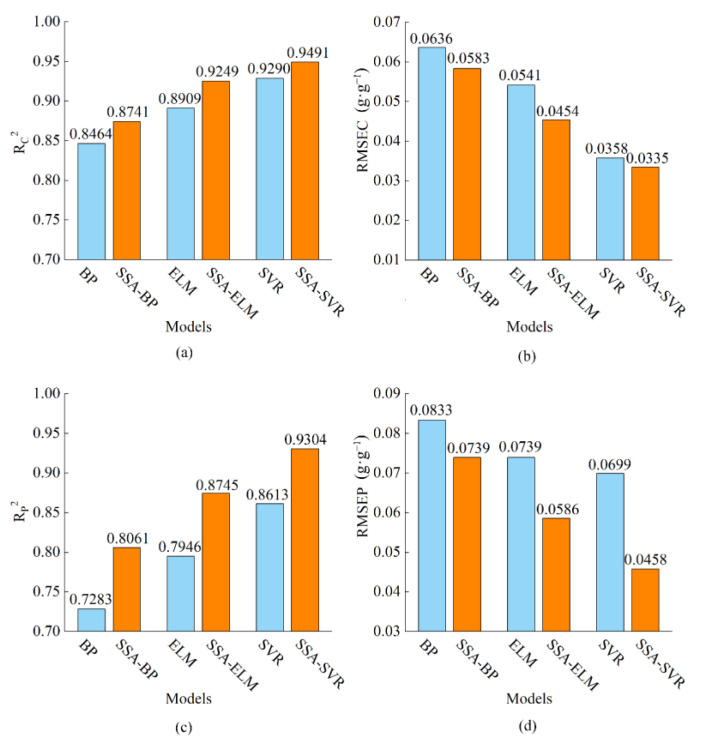
Comparison of R^2^ and RMSE for different models in each dataset. (**a**,**b**) Calibration set (10-fold cross-validation); (**c**,**d**) prediction set. R_C_^2^: coefficient of determination in calibration set; R_P_^2^: coefficient of determination in prediction set; RMSEC: root mean squared error for calibration set; RMSEP: root mean squared error for prediction set.

It can be seen from Figure 5 that SSA optimisation for models improves the performance of each data set compared with the use of BP, ELM and SVR models alone. Compared with those of the BP model, the R^2^ of the calibration set and prediction set of the SSA-BP model for quantification increased by 0.0277 and 0.0778 and RMSE decreased by 0.0053 g·g^−1^ and 0.0094 g·g^−1^, respectively. Compared with those of the ELM model, R_C_^2^ and R_P_^2^ of the SSA-ELM model increased by 0.0340 and 0.0799, whilst RMSEC and RMSEP decreased by 0.0088 g·g^−1^ and 0.0153 g·g^−1^, respectively. Compared with those of the SVR model, R_C_^2^ and R_P_^2^ of the SSA-SVR model increased by 0.0201 and 0.0691, whilst RMSEC and RMSEP decreased by 0.0024 g·g^−1^ and 0.0241 g·g^−1^, respectively. These results showed that the quantification performance of all models improves when SSA optimisation is applied and SSA-SVR achieves the optimal quantification performance.

**Figure 6:**
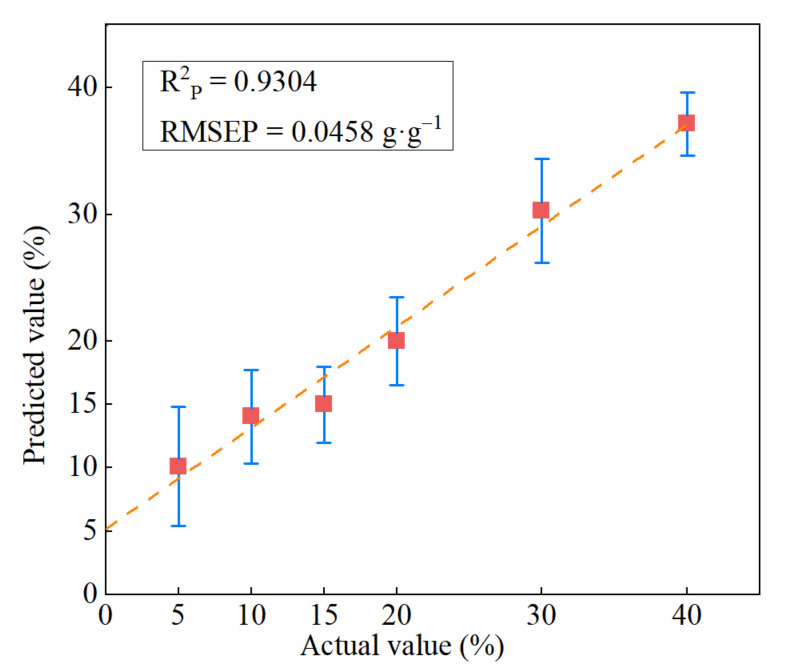
The result in prediction set of the SSA-SVR with error bar.

The results in the prediction set of the SSA-SVR with error bar are illustrated in Figure 6. The prediction standard deviation was utilised to reflect the degree of deviation between the predicted value and actual value. It could be seen that all predicted values were above 0% and were distributed near the regression line. This finding showed that all adulteration mixture samples in different proportions were effectively predicted by SSA-SVR [21]. Although the addition of mutton flavour essence changed odour information and effected sensory testing, NIR-HSI combined with the SSA-SVR model can effectively predict the proportion of pork mixed in mutton.

## 4. Conclusions

In this research, near-infrared hyperspectral imaging combined with machine learning and sparrow search algorithm was used to classify and quantify adulterated mutton with pork under the effect of mutton flavour essence. Classification models using BP, ELM and SVM were developed to discriminate mutton, pork and adulterated mixture samples. Quantification models using BP, ELM and SVR were developed to predict different proportions of pork in mutton. SSA was used to optimise the model parameters and the model results improved after optimisation. The results showed that the optimal classification model is SSA-SVM, with an accuracy of 99.79% in the prediction set, the optimal quantification model is SSA-SVR with an R_P_^2^ of 0.9304 and an RMSEP of 0.0458 in the prediction set. The results indicated that the NIR-HSI combined with ML and SSA had the remarkable ability to identify adulterated mutton with pork under the effect of mutton flavour essence. The rapid, non-destructive and automatic method proposed in this study can improve the detection efficiency, reduce the detection cost in practical application and has good application prospects. This study can provide a theoretical and practical reference to the evaluation and supervision of food quality under complex conditions, such as diversified adulteration and multiple food additives.

## Figures and Tables

**Table 1:** The discrimination results in each data set using the basic models and the optimisation models.

Models	Calibration Set (10-Fold Cross-Validation)	Prediction Set
	Accuracy (%)	Accuracy (%)
BP	92.92%	91.67%
ELM	97.15%	90.83%
SVM	99.65%	99.37%
SSA-BP	93.47%	92.50%
SSA-ELM	99.44%	98.13%
SSA-SVM	99.90%	99.79%

Note: BP: back propagation neural network; ELM: extreme learning machine; SVM: support vector machine; SSA: sparrow search algorithm.

**Table 2:** The prediction results in each data set using the basic models and the optimisation models.

Models	Calibration Set (10-Fold Cross-Validation)		Prediction Set	
	R_C_^2^	RMSEC (g·g^−1^)	R_P_^2^	RMSEP (g·g^−1^)
BP	0.8464	0.0636	0.7283	0.0833
ELM	0.8910	0.0542	0.7946	0.0739
SVR	0.9290	0.0359	0.8613	0.0699
SSA-BP	0.8741	0.0583	0.8061	0.0739
SSA-ELM	0.9249	0.0454	0.8745	0.0586
SSA-SVR	0.9491	0.0335	0.9304	0.0458

Note: R_C_^2^: coefficient of determination in calibration set; R_P_^2^: coefficient of determination in prediction set; RMSEC: root mean squared error for calibration set; RMSEP: root mean squared error for prediction set; SVR: support vector regression.

## Data Availability

The data presented in this study are available on request from the corresponding author.

## Supplementary Materials

The following supporting information can be downloaded at: https://www.mdpi.com/article/10.3390/foods11152278/s1, Figure S1: Average spectra of different classes preprocessed with 1D + MSC; Figure S2: Average spectra of different proportions preprocessed with 1D + MSC.
