# Supplementary figures and images for: Evaluation of Mutton Adulteration under the Effect of Mutton Flavour Essence Using Hyperspectral Imaging Combined with Machine Learning and Sparrow Search Algorithm
Source: Foods. 2022 Jul 30;11(15):2278. doi: 10.3390/foods11152278 (PMC9368686; doi:10.3390/foods11152278)

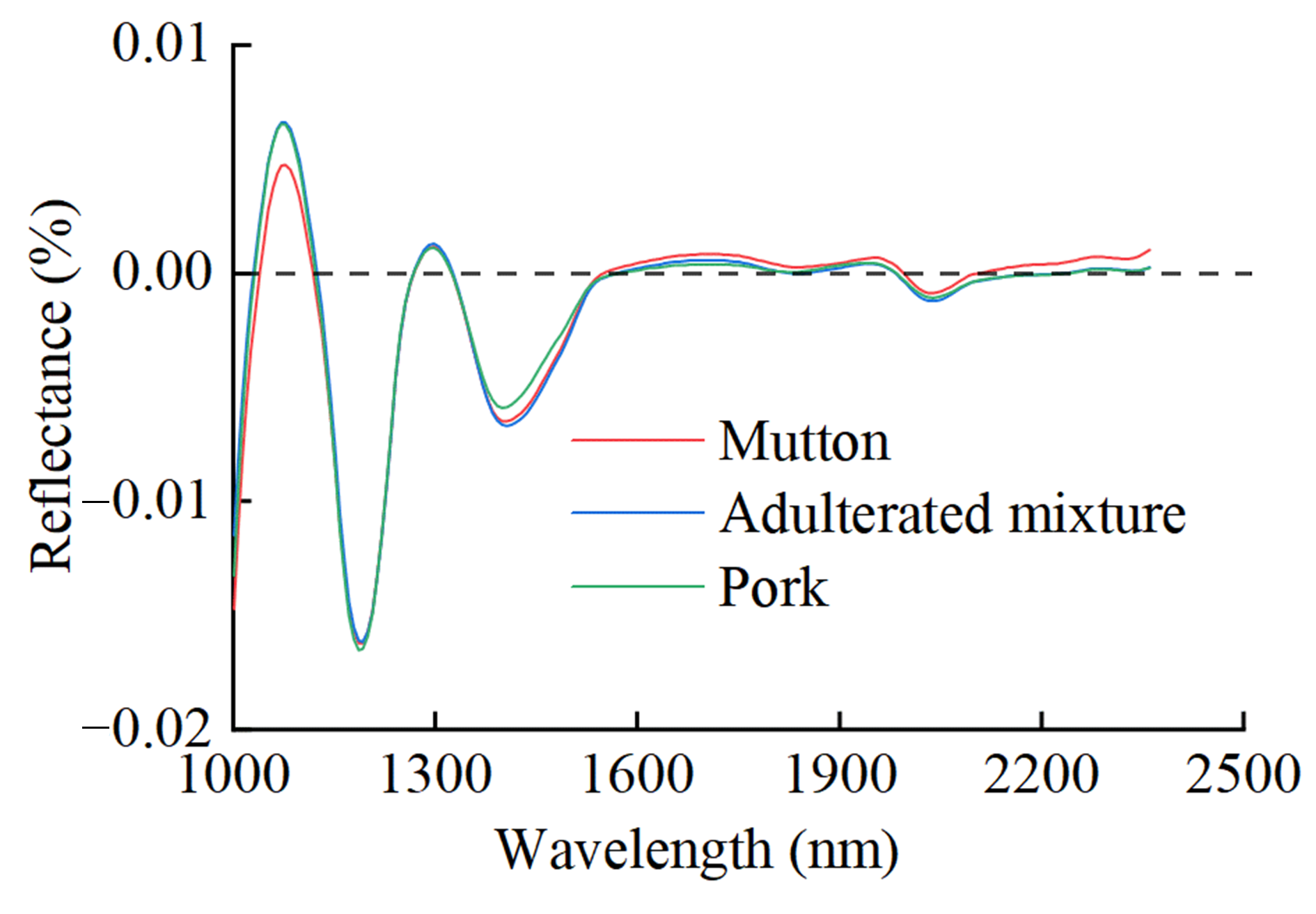

Supplement: Supplementary file 1 [file foods-11-02278-s001.zip › Figure S1.tif]

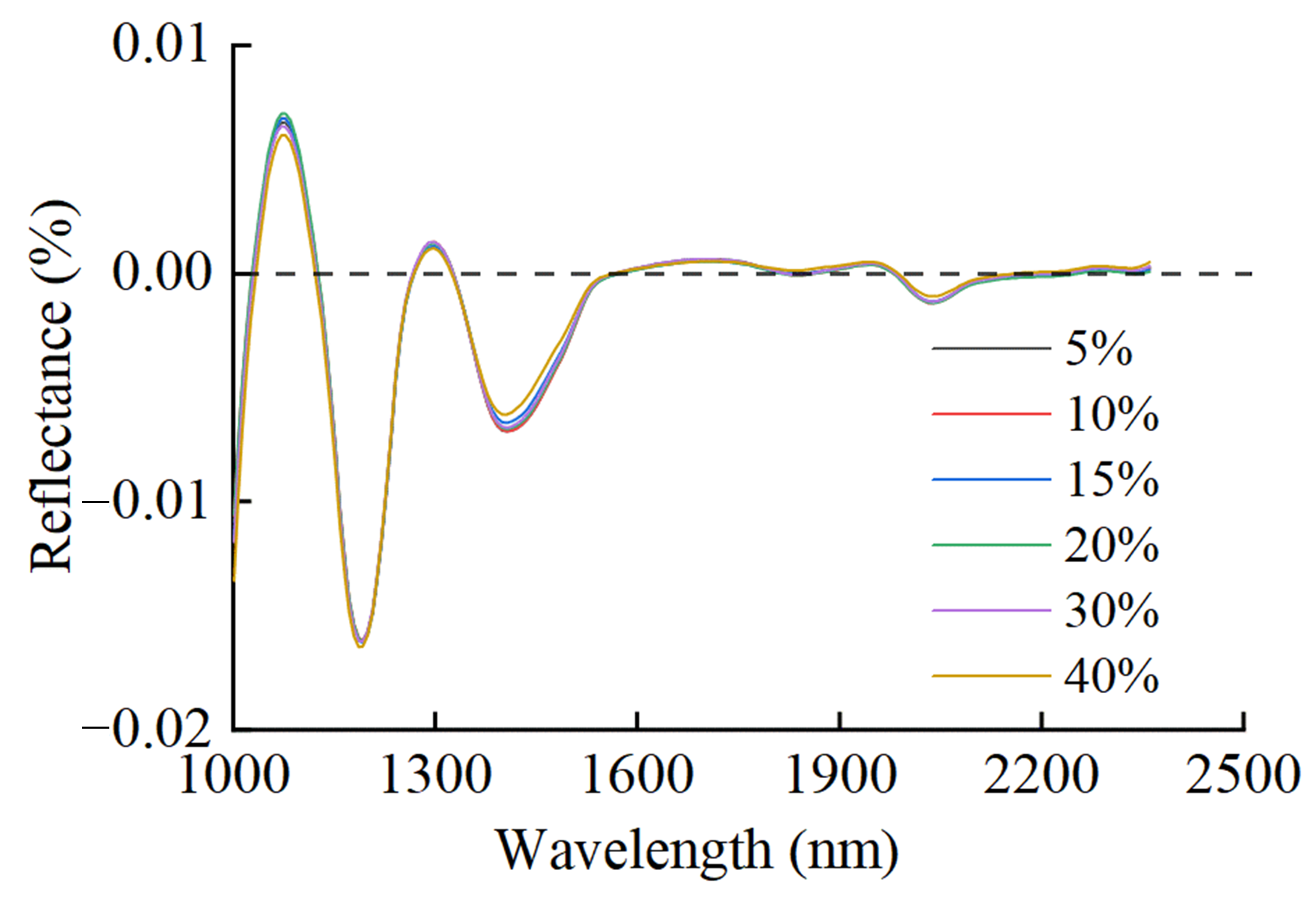

Supplement: Supplementary file 1 [file foods-11-02278-s001.zip › Figure S2.tif]
